# A systems biology approach for studying *Wolbachia* metabolism reveals points of interaction with its host in the context of arboviral infection

**DOI:** 10.1371/journal.pntd.0007678

**Published:** 2019-08-30

**Authors:** Natalia E. Jiménez, Ziomara P. Gerdtzen, Álvaro Olivera-Nappa, J. Cristian Salgado, Carlos Conca

**Affiliations:** 1 Centre for Biotechnology and Bioengineering (CeBiB), Department of Chemical Engineering, Biotechnology and Materials, University of Chile, Santiago, Chile; 2 Center for Mathematical Modeling (CMM) (UMI CNRS 2807), Department of Mathematical Engineering, University of Chile, Santiago, Chile; University of Queensland, AUSTRALIA

## Abstract

*Wolbachia* are alpha-proteobacteria known to infect arthropods, which are of interest for disease control since they have been associated with improved resistance to viral infection. Although several genomes for different strains have been sequenced, there is little knowledge regarding the relationship between this bacterium and their hosts, particularly on their dependency for survival. Motivated by the potential applications on disease control, we developed genome-scale models of four *Wolbachia* strains known to infect arthropods: *w*AlbB (*Aedes albopictus*), *w*VitA (*Nasonia vitripennis*), *w*Mel and *w*MelPop (*Drosophila melanogaster*). The obtained metabolic reconstructions exhibit a metabolism relying mainly on amino acids for energy production and biomass synthesis. A gap analysis was performed to detect metabolic candidates which could explain the endosymbiotic nature of this bacterium, finding that amino acids, requirements for ubiquinone precursors and provisioning of metabolites such as riboflavin could play a crucial role in this relationship. This work provides a systems biology perspective for studying the relationship of *Wolbachia* with its host and the development of new approaches for control of the spread of arboviral diseases. This approach, where metabolic gaps are key objects of study instead of just additions to complete a model, could be applied to other endosymbiotic bacteria of interest.

## Author summary

The expansion of the geographic distribution of arthropods has led to an increase in the number of infections of Zika and Dengue. This motivates the search for new alternative approaches for disease control. *Wolbachia pipientis*, an obligate intracellular bacteria known to provide pathogen-blocking capabilities to its host, has been used to this end. *Wolbachia*-infected mosquitoes have been released into the environment as a strategy for controlling the mosquito population and hence the spread of arboviral-derived diseases. However, there is little knowledge regarding the specific interactions that occur between *Wolbachia* and its host, particularly those associated with its obligate intracellular nature. In this work we studied *Wolbachia* endosymbiosis from a systems biology perspective. By analyzing the gaps in the metabolic networks of four *Wolbachia* strains we were able to identify key interaction points between this intracellular bacteria and its host, which play a crucial role in their metabolic relationship. This approach focuses on what is missing to allow unveiling new information regarding the interplay between interacting metabolic networks, and it is especially useful in the case of intracellular bacteria where experimental information is scarce. This additional insight on the metabolic interactions between host and parasite hold great potential for the development of arboviral disease control strategies.

## Introduction

*Wolbachia* are obligate intracellular alpha-proteobacteria, member of the Rickettsiales order known to infect nematodes and arthropods by developing diverse complex interactions with their hosts, such as supplementation with vitamins, cytoplasmic incompatibility and parthenogenesis [1–4].

The nature of these interactions is influenced by the strain and organism involved and they have been reviewed extensively [4]. In some organisms, *Wolbachia* has shown to impart a fitness advantage to arthropod host such as better reproductive traits or improved resistance to virus infection [5]. Particularly, interactions between this endosymbiont with its hosts range from metabolite supplementation, particularly biotin [6], riboflavin [7], to pathogenic interactions such as those of the strain *w*MelPop [8].

Several studies have been developed towards the understanding of *Wolbachia* interactions with their hosts driven by potential applications in development of novel control strategies for the spread of arbovirus-derived diseases such as Yellow fever, Zika, Chikungunya and Dengue [9]. Genomes of several *Wolbachia* strains have been sequenced [8,10–12] and comparatively analyzed [8] in order to explain host-symbiont features of interest such as cytoplasmic incompatibility.

*Wolbachia* are known to have a reduced genome size as a result of their adaptation to depend on other organism to their survival. Thus, it is expected that they exhibit a small and rather incomplete metabolic network, as it has been previously observed for other endosymbiotic bacteria [13,14]. We hypothesized that a thorough analysis of *Wolbachia* metabolism could be achieved by analyzing a representation of their wide metabolic capabilities given by genome-scale models.

Genome-scale models (GEMs) have emerged as a powerful tool for studying cellular metabolism since they provide a global representation of all biochemical transformations that could be carried out by a specific organism based on their genome annotation [15].

In this work, we developed genome-scale models for four *Wolbachia* strains found in insect arthropods, *w*AlbB (from *Aedes albopictus*) [11], *w*VitA (from *Nasonia vitripennis*) [10], *w*Mel [12] and *w*MelPop [8] (both from *Drosophila melanogaster*). We hypothesized that the analysis of the metabolic gaps in the curation stage could reveal potential candidates to explain the endosymbiotic nature of *Wolbachia* as shared metabolites between both species.

## Materials and methods

Four strains of *Wolbachia pipientis* were selected based on their characteristics, such as: their ability of causing cytoplasmic incompatibility, to infect mosquito species and pathogenicity. *w*AlbB (*Aedes albopictus*), *w*VitA (*Nasonia vitripenni*s) and *w*Mel and *w*MelPop (*Drosophila melanogaster*) genomes were used to generate draft genome-scale models using modelSEED [13]. Gap filling was performed considering a complete media extracellular environment, which means that the model can consume any nutrient for which a transport reaction is available to the model. This gap filling was analyzed to determine potential gene gaps which could putatively explain *Wolbachia pipientis*' symbiotic relationship with its hosts.

We proposed a draft biomass composition for *Wolbachia* based on phylogenetic information and predicted pathways in the annotation process [13]. The obtained composition was modified based on DNA and amino acid composition and updated fatty acid content based on reported concentration of phospholipids in *Wolbachia*, *Rickettsia* and *Escherichia coli* [16–19]. Computation of the stoichiometric coefficients associated with each component in the biomass function was achieved based on the protocol proposed by Thiele et al., (2010) [15].

Integration of the four obtained *Wolbachia* metabolic reconstructions was achieved using COBRApy [20]. Gene protein reaction (GPR) associations are modified to integrate gene identifiers of all the studied strains. The obtained gaps were confirmed using BlastP to detect top hits to unannotated gene products, which were selected and incorporated into the genome-scale model when E-values were lower than 0.001 and coverage was above 85%. Flux Balance Analysis (FBA) simulations were achieved using COBRApy [20].

## Results

Four *Wolbachia* strains were selected based on the following characteristics: *w*VitA due to its reported ability to cause cytoplasmic incompatibility, *w*AlbB was known to infect the mosquito *Aedes albopictus*, *w*Mel due to its reported effects on pathogen virus blocking and *w*MelPop because of its pathogenicity to their host. Based on their genome annotation, four genome-scale models were obtained (Table 1, S1 File, S2 File).

**Table 1:** *Wolbachia pipientis* genome-scale models.

	*w*AlbB	*w*VitA	*w*Mel	*w*MelPop
**Genes**	324	334	316	329
**Reactions**	756	752	766	775
**Metabolites**	830	821	840	849

**Fig 1:**
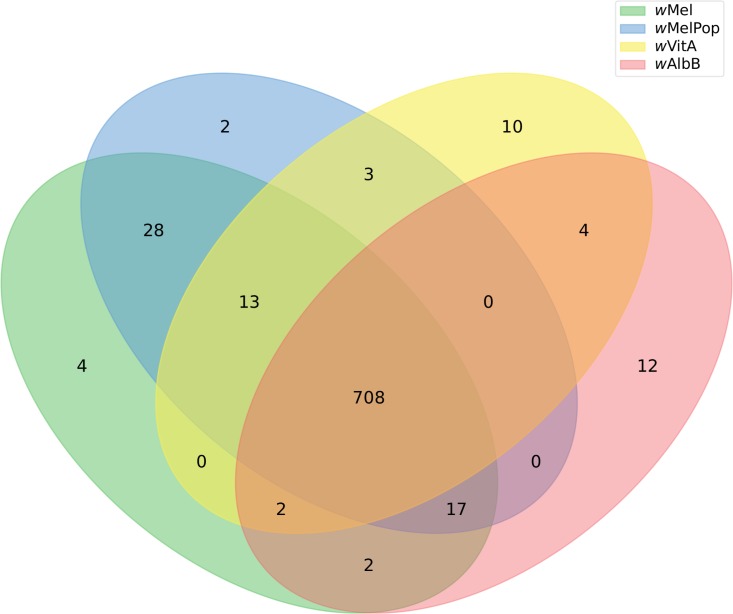
*Wolbachia* metabolic reconstructions. Reactions shared amongst the *Wolbachia* metabolic reconstructions for the strains causing cytoplasmic incompatibility (*w*Mel, *w*VitA), pathogen blocking (*w*Mel and *w*MelPop), the pathogenic strain *w*MelPop, and the *Aedes albopictus* strain *w*AlbB.

All four *Wolbachia* metabolic reconstructions share 708 common reactions. Strains *w*AlbB and *w*VitA have 4 reactions in common which are absent in *w*Mel and *w*MelPop, *w*VitA has 10 unique reactions and *w*AlbB includes 12 reactions that are not present on the model of the strain that infects other hosts. On the other hand, *w*Mel and *w*MelPop have 28 common reactions that are not present in the other two strains, while *w*Mel includes 4 unique reactions and *w*MelPop 2 unique reactions of their own (Fig 1).

*w*VitA presents 10 unique reactions not shared with the other 3 analyzed strains, including *w*Mel and *w*AlbB which are also known to induce cytoplasmic incompatibility. These reactions are associated with the use of deoxyguanosine as a precursor of dGTP and thioredoxin oxidation and are not associated with this phenotype. Cytoplasmic incompatibility has been studied and associated with two genes: *cifA* and *cifB* in *Drosophila melanogaster* [21] which have been associated with regulatory processes that are not represented by this approach.

On the other hand, *w*AlbB has 12 reactions exclusive to this strain involving protocatehuic acid (PCA), a component known to be part of the protein fraction of the insect cuticule and *w*MelPop includes 2 unique reactions associated with tetrahydrofolate metabolism, which are not associated with its pathogenic nature and present gaps of its metabolic nature. We then propose that *w*MelPop detrimental effect on its host lies in regulatory processes that determine cell density inside its host rather than in specific metabolic reactions that could be associated with pathogenicity.

### *Wolbachia* metabolism

We analyzed previously reported metabolic features for the *w*Mel strain, which has been described to rely mainly on amino acids to support their energetic requirements, having limited carbohydrate synthesis capacity and being unable to transport ATP directly from its host and to synthesize Lipid A [12,22]. Our findings support these affirmations, with a *Wolbachia* metabolic reconstruction exhibiting a complete glycolysis pathway starting from fructose 6 phosphate towards phosphoenolpyruvate and a complete TCA (tricarboxylic acid) cycle.

Additionally, *Wolbachia* presents a highly conserved pentose phosphate pathway (PPP) for sugar nucleotide synthesis, contrary to what has been reported for close organisms such as *Rickettsia* [14] and an amino acid metabolism, characterized by the presence of amino dipeptidases. This suggests that dipeptide transport is required by this endosymbiont for acquiring amino acids derived from its host metabolism.

The riboflavin synthesis pathway, another putative symbiosis-determining pathway, is highly conserved with one missing step that is common to all studied strains, FMN hydrolase/5-amino-6-(5-phospho-D-ribitylamino) uracil phosphatase. However, this reaction has been found to be associated with the gene *ribD* reported to be found in *w*Mel, *w*Ri, *w*Ha, and *w*Au [7]. Therefore, this is not an incomplete metabolic pathway in any of the studied strains.

We also analyzed the biomass reaction given by modelSEED which is generated from phylogenetic information and the predicted pathways based on their genome annotation [13]. Particularly, this stoichiometric composition is derived from the Gram-negative bacteria template and the presence of predicted metabolic pathways such as ubiquinone biosynthesis, fatty acid biosynthesis, and polyamine metabolism among others.

However, *Wolbachia* is closer to the *Rickettsia* genus rather than *Escherichia coli*, the exemplary Gram-negative species. Subsequently, the biomass composition was modified to include specific phospholipid and fatty acid composition derived from studies made in *Escherichia coli* and particularly in *Rickettsia prowazekii* [16–19], in order to find specific network spots where the symbiotic nature of the predicted gaps in this metabolic reconstruction could be found.

### Gap analysis

We performed an analysis of the gap filling process to determine candidates for the metabolic relationship between *Wolbachia* and its hosts (S3 File). In this analysis all four metabolic reconstructions shared the same 127 gap fill reactions (75%) which are presumably requirements for synthesis of biomass components that no *Wolbachia* strain is able to perform.

The added reactions correspond approximately to 20% of the total number of reactions in the analyzed metabolic reconstructions. This high proportion as a single fact putatively explains single handedly the obligate endosymbiotic nature of *Wolbachia*, as it has been discussed for other organisms of similar nature [13].

**Fig 2:**
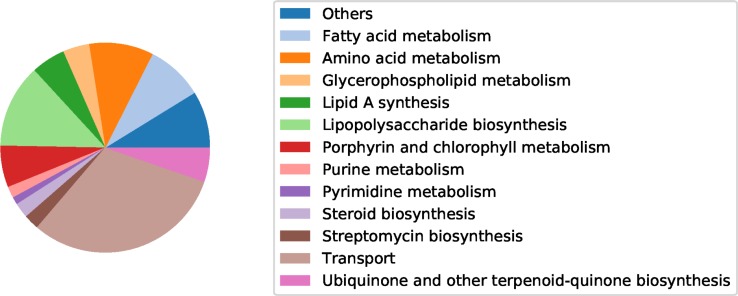
Metabolic gaps in *Wolbachia pipientis*. Functionality of the metabolic gaps in the obtained *Wolbachia* metabolic reconstruction.

Most of these reactions are associated with transport of essential metabolites for cell growth instead of *de novo* synthesis since internalization of external metabolites requires the addition of a lower number of reactions to the model, a feature that was preferred in our reconstruction framework **(**Fig 2). However, it is worth mentioning that transport reactions are only about one third of the gap reactions of the model, which means that real gaps are also present in our reconstructions that are not solved by importing metabolites into the cell.

An inspection of the added reactions for *w*AlbB and *w*VitA showed that potential metabolic candidates for explaining their symbiotic relationship with their hosts are mainly associated with fatty acid, lipopolysaccharide and amino acid metabolism. Arginine, glutamine and alanine are particularly important for *w*AlbB and asparagine, glutamine, histidine, isoleucine and leucine for *w*VitA. Additionally, *w*AlbB requires transport of TTP, hexadecanoate and dGTP for survival while *w*VitA requires transport of histidine and CTP.

*w*Mel and *w*MelPop share over 90% of their metabolic reactions, which is expected due to their phylogenetic closeness. An analysis on these reactions showed that biosynthesis of antibiotic precursors, lipopolysaccharide biosynthesis and alanine, serine and glycine metabolism are metabolite candidates to explain *Wolbachia* interaction with its hosts.

*Wolbachia pipientis* is known to be highly dependent on amino acid metabolism of its hosts [12], which is consistent with the obtained gaps in the analyzed strains. In support of this, transport of dipeptides and amino acids has been added in the gap filling process, which can be another factor to explain the symbiotic relationship of *Wolbachia* with their hosts.

The obtained metabolic reconstruction also includes gaps associated with lipid A synthesis, which has previously been hypothesized to be absent in *w*Mel and *w*Bm [22]. In fact, transformations of amino acids initially found to be gaps in this model are part of the synthesis of this precursor of lipopolysaccharide (LPS) synthesis.

**Fig 3:**
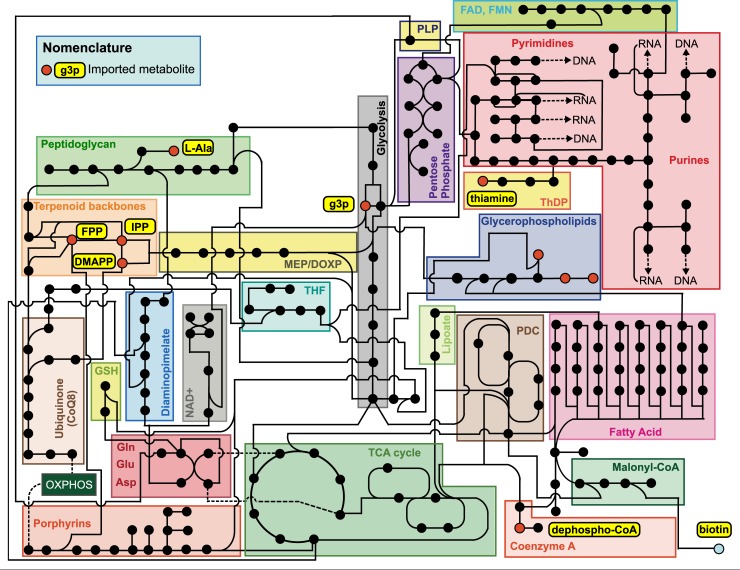
Wolbachia metabolism. *Wolbachia* presents highly conserved pathways for nucleotide, peptidoglycan and fatty acid synthesis but depends highly on transport of carbon sources such as glycerate 3 phosphate, amino acids such as alanine, and phospholipid transport to support cell growth. g3p: glycerate 3 phosphate, FPP: farnesyl pyrophosphate, IPP: isopentenyl phosphate, DMAPP: dimethylallyl pyrophosphate. Adapted from [14].

Given the phylogenetic closeness of genera *Wolbachia* and *Rickettsia*, the obtained metabolic gaps for the different *Wolbachia* strains were compared to the ones reported for *Rickettsia* [14]. An analysis showing metabolic features of *Wolbachia pipientis* and predicted imported metabolites derived from gap analysis of this genome-scale model (Fig 3**)** that although both are members of the Anaplasmataceae family, the reductive genome evolution has led to the loss of different functionalities in each phylogenetic branch.

*Rickettsia* shows depleted metabolic pathways for B vitamins, several cofactors and the pentose phosphate pathway [14], contrary to what has been obtained in this work, where these metabolic pathways are highly conserved (Fig 3**)**. The obtained metabolic reconstruction is able to synthesize S-adenosyl methionine (SAM) from methionine but includes gaps associated with SAM metabolization for heme synthesis, which was initially reported to also be missing in the nematode infecting strain *w*Oo [23] but then experimentally found to be fully present in *Wolbachia* [24].

On the other hand, common metabolic gaps between *Wolbachia* and *Rickettsia* have also been identified. The obtained *Wolbachia* model has reactions without associated genes involved in the synthesis of isopentenyl phosphate (IPP) and 4-hydroxybenzoate from lanosterol (S2 and S3 Files) while *Rickettsia* is known to import IPP and 4-hydroxybenzoate so we infer that *Wolbachia* must also acquire these metabolites from its host. Tetrahydrofolate has also been proposed as an imported metabolite in *Rickettsia*, which is consistent with our findings for *Wolbachia* in the analysis of *Wolbachia*'s metabolism.

## Discussion

*Wolbachia* are endosymbiotic bacteria of interest due to their interactions with their arthropod hosts, such as pathogen blocking, which includes blocking of human arboviral pathogen spread, and cytoplasmic incompatibility. In this work we present metabolic reconstruction of four *Wolbachia* strains known to infect arthropods, *w*Mel, *w*MelPop, *w*VitA and *w*AlbB. An analysis of these metabolic reconstructions is focused on their metabolic gaps as these missing reactions may be the key interactions with its hosts that explain their obligate endosymbiont nature and the difficulty found to cultivate these bacteria in the laboratory outside their host cell.

*Wolbachia* are known to have a metabolism that is highly dependent on amino acids for cell growth [12,25]. The presence of amino peptidases suggests that this endosymbiont is able to feed on peptides derived from proteolytic processes inside their host. The obtained metabolic reconstruction predicts the lack of genes associated with lipid A synthesis as it has been proposed for the strain *w*Mel and *w*Bm [22].

A series of confirmed gaps are associated with IPP and 4-hydroxybenzoate synthesis which are metabolic requirements for ubiquinone 8 synthesis, a highly conserved pathway in *Wolbachia*. Since these metabolites have been previously predicted to be imported in *Rickettsia*, we propose that similar mechanisms exist for their import in this alpha-proteobacteria.

Regarding our proposed methodology to find metabolites linked to the endosymbiotic nature of *Wolbachia*, several factors are known to affect the gap filling process in genome-scale models, such as media and biomass composition. Different extracellular environments result into different sets of metabolites that could be added, as transport reactions, into the intracellular compartment of the obtained model. On the other hand, the artificial inclusion of components that are not part of the modeled organism's biomass, result in the addition of metabolic capabilities observed to be absent experimentally.

In this work, gap analysis was performed based on the obtained models for four different *Wolbachia* strains. The media composition was assumed to be rich in nutrients due to the obligate intracellular location of *Wolbachia*. Additionally, the biomass function was updated to represent the experimentally determined *Wolbachia* phospholipid and fatty acid content. The actual existence of the obtained gaps was confirmed by searches using BlastP against non-redundant protein and translated nucleotide databases. Their essentiality was assessed based on the upgraded *Wolbachia* biomass composition, as well as the composition of their surrounding environment to guarantee an improved quality of the predictions made by this approach.

Alternative strategies to study metabolism of endosymbionts have been published recently. Driscoll et al., (2017) [14] reconstructed *Rickettsia* metabolism and predicted transported compounds based solely on observed gaps in their metabolic network without information on the requirements for cell growth given by their biomass composition. *Rickettsia* exhibits depleted metabolic pathways for B vitamins, and the pentose phosphate pathway contrary to our findings in *Wolbachia*, which presents highly conserved pathways for synthesis of vitamins and nucleotides. These differences can be associated with the endosymbiotic and rather mutualistic behavior of *Wolbachia* versus *Rickettsia* which has a pathogenic nature. Accordingly, *Rickettsia* presents a metabolism oriented to the import of metabolites (SAM, ATP) whilst *Wolbachia* conserves metabolic pathways for metabolic provisioning to its hosts.

There is no available automatic method to distinguish between true annotation gaps, which have biological meaning and correspond to gene losses that explain the dependency of *Wolbachia* on its host for their survival, from annotation gaps originated from experimental errors in the process of sequencing and annotation of the DNA of the analyzed strains. Particularly, of the 1,174 genes found originally in the annotation process for *w*Mel [12], only 316 were linked to metabolic functions represented in our genome-scale model, showing that although there is valuable information regarding metabolic interactions derived from *Wolbachia* gene annotation, there is a considerable number of genes associated with regulatory processes that are not represented by this approach. Widely studied *Wolbachia* genes as the cytoplasmic incompatibility genes (*cifA*, *cifB*) [21] and ankyrin repeat proteins [12] are not included in genome-scale models, given that they are not associated with chemical reactions that this organism can carry out.

We propose that the inclusion of information of multiple strains could help minimize this problem and provide the scientific community with a robust tool for metabolic studies of this intracellular bacteria. Additionally, this approach, where metabolic gaps are key objects of study instead of just additions to complete a model, could be expanded for other endosymbiotic bacteria of interest.

## Conclusion

In this work we present *Wolbachia* genome-scale model for four strains known to infect arthropods. We propose that the metabolic gaps present in this model are key to study metabolic interactions with their hosts and could potentially influence processes that lead to cytoplasmic incompatibility and pathogen blocking.

*Wolbachia* metabolism is characterized by the import of amino acids for energy and growth, and also particularly the import of isopentenyl phosphate and 4-hydroxybenzoate for ubiquinone 8 biosynthesis. Although, *Wolbachia* share several characteristics with *Rickettsia*, the metabolism of the analyzed *Wolbachia* strains presents highly conserved pathways for nucleotide synthesis and riboflavin, which can be ascribed to their mutualistic rather than parasitic behavior.

We have provided a systems biology analysis framework to study *Wolbachia* metabolism to improve the understanding on the relationship with its host and the development of new approaches towards the control of spread of arboviral diseases.

## Supporting information

S1 File *Wolbachia* genome-scale models. *Wolbachia* genome-scale models in SBML for the four analyzed strains *w*Mel, *w*MelPop, *w*AlbB, and *w*VitA.
(ZIP)

S2 File *Wolbachia* metabolism and gene associations. Spreadsheet (xlsx format) listing all the included reactions and gene associations for the four *Wolbachia* genome-scale models.
(XLSX)

S3 File New annotated genes and gaps. Spreadsheet (xlsx format) listing new annotated genes and filled metabolic gaps derived from this work.
(XLSX)
